# Letrozole is superior to clomiphene citrate in ovulation induction in patients with polycystic ovary syndrome

**DOI:** 10.12669/pjms.36.7.3345

**Published:** 2020

**Authors:** Mehmet Nafi Sakar, Suleyman Cemil Oglak

**Affiliations:** 1 Mehmet Nafi Sakar, Department of Obstetrics and Gynecology, Memorial Diyarbakir Hospital, Diyarbakır, Turkey; 2 Suleyman Cemil Oglak, Department of Obstetrics and Gynecology, University of Health Sciences, Gazi Yasargil Training and Research Hospital, Diyarbakır, Turkey

**Keywords:** Polycystic ovary syndrome, Ovulation induction, Clomiphene citrate, Letrozole

## Abstract

**Objective:**

This study was aimed to compare the clinical outcomes of ovulation induction (OI) by timed intercourse with letrozole (LTZ) and clomiphene citrate (CC).

**Methods:**

Three hundred and twenty-three patients with polycystic ovary syndrome (PCOS) who underwent OI with LTZ or CC between February 2017 and November 2018 were included in this retrospective study. The patients were divided into two groups as the CC group (n=148) and the LTZ group (n=175). Endometrial thickness, follicular development, ovulation, clinical pregnancy, abortion, and live birth rates of the groups were analyzed.

**Results:**

The mean endometrium thickness of the CC group was 7.1±1.7 mm, and the LTZ group was 8.6±1.8 mm (p<0.001). The ovulation rate per cycle was higher in the LTZ group (93.1%) in comparison with the CC group (83.8%) (p=0.013). Clinical pregnancy rates were 52% in the LTZ group, and 41.2% in the CC group (p=0.047). LTZ with 44% of live birth rate was superior to CC with a 33% live birth rate (p=0.029).

**Conclusions:**

LTZ is an effective OI agent in PCOS patients. LTZ is superior to CC in terms of pregnancy rates and live birth rates. As a result, we recommend that LTZ should be the first-line treatment agent in patients with PCOS.

## INTRODUCTION

Polycystic ovary syndrome (PCOS) is the most common cause of anovulatory infertility.^1^ Although the prevalence in community studies is between 6-21%, this rate reaches 70% in anovulatory infertility.^1^,^2^ The Rotterdam criteria continue to be discussed in certain respects, but these criteria are still used in the diagnosis of PCOS.^3^

Clomiphene citrate (CC) is advised to be the first drug of choice for the ovulation induction (OI) of PCOS patients since it is safe and effective, readily available, low-cost, and well-tolerated.^4^ CC, a selective estrogen receptor modulator (SERM), binds nuclear estrogen receptors for an extended period and modulates the function of estrogen.^5^ CC inhibits the negative feedback of estrogen and stimulates the release of gonadotropin-releasing hormone (GnRH) in the hypothalamus, resulting in stimulation in the secretion of pituitary gonadotropins, and thus induces ovarian follicular activity.^6^ However, CC has disadvantages in treatment. About 20-25% of patients show no response to CC and are considered to be CC resistant.^7^ CC depletes estrogen receptors and has a long half-life (2 weeks), causing adverse effects on endometrial thickness and cervical mucus.^2^,^8^ This effect leads to a pregnancy rate of approximately 18% despite the high ovulation rate with CC.^9^

Letrozole (LTZ), an aromatase inhibitor, impedes the estrogen production by inhibiting the aromatase in the ovary and release the hypothalamic-pituitary-ovarian (HPO) axis from the negative feedback of estrogen. The increased release of gonadotropins promotes ovarian follicle growth and stimulates ovulation.^10^ Also, LTZ increases intraovarian androgens. Androgens have an essential role in early follicular development by augmenting follicle-stimulating hormone (FSH) receptors and stimulating insulin-like growth factor-I (IGF-I). FSH and IGF-I perform synergistically to improve follicular growth.^8^ Unlike CC, LTZ has no anti-estrogenic effects and has a short (45 hour) half-life.^11^ Due to these effects, LTZ does not cause an adverse effect on cervical mucus and endometrial thickness, and thus pregnancy rates are higher.^12^ Also, the rate of mono-follicular development and singleton pregnancy rates are higher in ovulation induction (OI) with LTZ.^13^ This study aimed to compare the clinical outcomes of OI by timed intercourse with LTZ and CC in PCOS patients.

## METHODS

This retrospective study included 323 patients with PCOS ranging in age from 20 to 35 years who admitted to Gazi Yaşargil Training and Research Hospital Infertility policlinic between February 2017 and November 2018. Patients between the ages of 20-35 years, diagnosed with PCOS according to Rotterdam criteria, proved to have patency of at least one side of the fallopian tube and normal uterine cavity with hysterosalpingography (HSG), and normal spermiogram parameters of their spouses were included in the study. According to the Rotterdam criteria, patients were diagnosed with PCOS if two of the following three criteria exist: Oligo/anovulation, clinical/biochemical hyperandrogenism, and polycystic ovary appearance on ultrasonography (US). Oligo/anovulation was defined as a menstrual pattern of oligo/amenorrhea (duration of cycle >35 days) and/or low mid-luteal serum progesterone concentration. Cause of infertility other than anovulation, previous history of ovarian surgery, previous history of exposure to cytotoxic drugs, a history of pelvic radiation therapy, patients with adult-onset congenital adrenal hyperplasia, Cushing syndrome, hypertension, hyperlipidemia, liver or kidney dysfunction, smokers and alcohol drinkers were excluded. Age, body mass index (BMI), duration of the marriage, duration of infertility, type of infertility (primary, secondary), basal FSH, basal luteinizing hormone (LH), basal estradiol (E2), prolactin, and thyroid-stimulating hormone (TSH) values were recorded. The Ethics Committee of Gazi Yaşargil Training and Research Hospital approved the study (19.01.2018/9).

This study is a retrospective comparative study of 323 infertile patients. CC or LTZ was used as standard protocols for OI in infertile patients with PCOS. Patients were informed entirely about the mechanism of effect and the experiential essence of LTZ. The off-label indication of LTZ was wholly explained. The use of LTZ was based on the couple’s choice. The patients were divided into two groups as the CC group (n=148) and the LTZ group (n=175). CC group received 50 mg of CC twice daily, and the LTZ group received 2.5 mg of LTZ twice daily. In all patients, OI was initiated after a transvaginal ultrasound (TVUS) examination between 2-5th days of the spontaneous menstrual cycle or withdrawal bleeding provided by 10 mg/day medroxyprogesterone acetate. CC or LTZ continued for five days. Five days after the last dose, patients were evaluated for endometrial thickness and the presence of follicles over 10 mm by TVUS. The presence of follicles over 10 mm was assessed in response to the medication. Serial TVUS examinations were continued until a mature follicle of diameter 18 mm was seen. The number of follicles and endometrial thickness of patients were recorded. None of the patients received human chorionic gonadotropin (hCG) to trigger ovulation. Ovulation was confirmed by determining the follicle collapse on following TVUS and measuring the level of serum progesterone. Serum progesterone levels >3 ng/mL were accepted as ovulation. Timed intercourse was recommended to patients with dominant follicles for one week every other day. OI continued until pregnancy occurs or for up to six cycles.

The pregnancy test was performed one week later in cases with confirmed ovulation. Pregnancy was considered positive if serum beta-hCG concentration was 5 IU/mL and above. TVUS was performed 1-2 weeks later. The gestational sac and/or fetal cardiac activity was accepted as clinical evidence of pregnancy. A decrease in serum beta-hCG concentrations following pregnancy test positivity was defined as biochemical pregnancy. Abortion was defined as clinical pregnancy loss. Births with >23 weeks and with cardiac beat were called live births. Endometrial thickness, follicular development, ovulation, clinical pregnancy, abortion, and live birth rates of the groups were analyzed.

### Statistical Analysis

IBM SPSS 21.0 for Windows (SPSS Inc., Chicago, IL, USA) statistical package program was used for statistical evaluation of our research data. Measured variables were presented as mean±standart deviation (std), and categorical variables were presented as numbers and percentages (%). Kolmogorov-Smirnov test was used to determine whether the numerical data matched the normality distribution. Mann-Whitney U test was used to compare the non-normally distributed data. A Chi-square test was used to compare qualitative variables. P-value <0.05 was considered statistically significant.

## RESULTS

During the study period, 148 patients who underwent OI with CC and 175 patients who underwent OI with LTZ were evaluated for their demographic characteristics, laboratory values, and clinical outcomes.

The demographic characteristics and laboratory values of the two groups are summarized in Table-I. There was no statistically significant difference between the groups in terms of age, BMI, primary/secondary infertility, duration of infertility, basal FSH, LH, prolactin, estradiol, and TSH levels.

**Table-I:** Demographic characteristics and laboratory values of the groups. mean±std, median [minimum-maximum]).

	Clomiphene Citrate group (n=148)		Letrozole group (n= 175)		P value

	Mean±std	Median (minmax)	Mean±std	Median (minmax)	
Age, years	24.6±4.4	24 (18-35)	25.9±4.0	25 (20-35)	0.506
BMI, kg/m^2^	24.8±2.9	24.2 (19.7-32.8)	25.4±3.2	25.0 (20.7-35.8)	0.075
Duration of infertility, years	2.5±2.3	2 (1-11)	2.7±2.1	2 (1-12)	0.077
Primary infertility, n(%)	90 (60.8)		100 (58.4)		0.672
Day 3 FSH, mIU/mL	5.3±1.4	5.12 (2.43-9.80)	5.7±4.4	5.0 (2.43-10.0)	0.697
Day 3 LH, mIU/mL	6.7±6.4	5.38 (1.7-25.1)	5.6±3.0	5.3 (1.52-20.0)	0.418
Day 3 estradiol, pg/mL	39.5±25.4	36.0 (4.3-226.0)	36.2±13.2	35.0 (6.8-86.0)	0.322
Prolactin, ng/mL	14.6±5.3	14.35 (5.8-31.0)	14.2±8.3	12.09 (3.2-59.8)	0.041
TSH	1.7±0.8	1.59 (0.5-4.4)	1.6±0.9	1.42 (0.6-7.3)	0.422

For statistical analysis, Kolmogorov-Smirnov and Mann-Whitney U tests were used.

The clinical outcomes of the groups following OI are summarized in Table-II. The mean endometrium thickness of the CC group was 7.1±1.7 mm, whereas the LTZ group was 8.6±1.8 mm, and this difference was statistically significant (p<0.001). The mean number of follicles ≥14 mm in the CC group (1.2±0.8) and the LTZ group (1.2±0.5) were similar (p=0.870).

**Table-II:** Clinical outcomes of the groups.

	Clomiphene Citrate group (n= 148)		Letrozole group (n= 175)		P value

	Mean±std	Median (minmax)	Mean±std	Median (minmax)	
Endometrium thickness, mm	7.1±1.7	6.7 (4.5-13.1)	8.6±1.8	8.0 (6.0-15.0)	<0.001***
Number of follicle ≥14mm	1.2±0.8	1 (0-3)	1.2±0.5	1 (0-2)	0.870
Mid-luteal serum progesterone, ng/mL	12.8±7.2	13.1 (0.9-40.0)	14.3±6.3	13.8 (1.2-35.0)	0.029*
Ovulation rates, n (%)	98 (66.2)		134 (76.5)		0.013*
Clinical pregnancy, n(%)	31 (20.9)		58 (33.1)		0.027*
Abortion rates, n (%)	5 (16.1)		7 (12.1)		0.804
Live birth, n (%)	26 (17.5)		51 (29.1)		0.023*
Fetal anomaly, n (%)	1 (3.2)		1 (1.7)		1.000

For statistical analysis, Kolmogorov-Smirnov, Chi-square, and Mann-Whitney U tests were used.

* p<0.05,

*** p<0.001

The mean mid-luteal serum progesterone value was 12.8±7.2 ng/mL in the CC group and 14.3±6.3 ng/mL in the LTZ group. This difference was statistically significant (p=0.029). The ovulation rate per cycle was higher in the LTZ group (76.5%) in comparison with the CC group (66.2%), and this difference was statistically significant (p=0.013). Out of 175 patients, 58 women (33.1%) became clinically pregnant in the LTZ group compared with 31 pregnant women (20.9%) out of 148 cases in the CC group, and this was statistically significant (p=0.027). The abortion rates of the CC group (16.1%) and the LTZ group (12.1%) were similar (p=0.804). When the groups were compared in terms of live birth rates, LTZ with 29.1% of live birth rate was superior to CC with a 17.5% live birth rate (p=0.023). In the CC group, there was one (3.2%) newborn with a cardiac anomaly, and in the LTZ group, there was one (1.7%) newborn with renal anomaly (p=1.000). Multiple pregnancies were not present in both groups. There were no significant differences between the two groups in terms of the gestational week at delivery, birth weight, and the newborn’s first and fifth minute Apgar score values (Table-III).

**Table-III:** Neonatal outcomes of the groups.

	Clomiphene Citrate group (n= 148)	Letrozole group (n= 175)	P value
Gestational week at delivery	37.8±2.7	38.2±2.6	0.446
Birthweight (g)	3028.0±362.2	3146±367.4	0.368
1st minute Apgar score	8.7±1.2	8.6±1.2	0.686
5th minute Apgar score	9.2±0.5	9.2±0.4	0.841

For statistical analysis, Kolmogorov-Smirnov and Mann-Whitney U tests were used.

## DISCUSSION

The current study demonstrated that OI with LTZ revealed higher ovulation, clinical pregnancy, and live birth rates with a significantly higher endometrial thickness than CC treatment in patients with PCOS.

Oral preparations are used as the first-line treatment in ovulation induction of patients with PCOS.^14^ Although CC has been used for a long time in treating patients with PCOS, the adverse effects it causes have led clinicians to seek different treatment modalities. For this purpose, LTZ is widely used, but the results are contradictory in studies comparing the success of CC and LTZ treatment in patients with PCOS.

Elevated BMI is an important clinical feature of PCOS and affects both the success of OI and can constitute a risk for adverse pregnancy outcomes.^15^ In our study, the mean BMI was 24.8±2.9 kg/m^2^ in the CC group and 25.4±3.2 kg/m^2^ in the LTZ group. These values were similar to the BMI values in other studies.^15^ Also, the patients included in the study were diagnosed with PCOS according to the Rotterdam criteria. Therefore, we suggest that this study group reflects a high similarity in patients with PCOS receiving infertility treatment. Also, there was no statistically significant difference between the two groups regarding demographic characteristics and laboratory values before OI. These results allow us to eliminate the predictors that may have a positive or adverse effect when comparing the efficacy of the two drugs.

In PCOS patients, follicle monitoring and timed intercourse have been shown to increase treatment success after initiation of OI.^16^ CC leads to depletion of estrogen receptors, and this effect is prolonged because of its long half-life.^17^ This depletion causes endometrial thinning and adversely changes the quality of cervical mucus. On the other hand, although LTZ creates an estrogen deficiency environment, it does not cause adverse effects on the endometrium due to its short half-life. Also, LTZ increases the expression of integrin, which is one of the endometrial receptivity markers.^17^,^18^ Baruah et al. showed that sub-endometrial blood flow was better in patients with PCOS treated with LTZ than CC.^19^ In another study, the endometrium was slightly thicker in the CC group than the LTZ group. In our study, the endometrium thickness was significantly higher in the LTZ group compared to the CC group. LTZ’s superior effect over CC in endometrial response enables OI success rates to increase.

LTZ reduces the estrogen level by inhibiting the aromatase enzyme, releasing the hypothalamus from the negative feedback effect of estrogen, and thereby increase FSH discharge.^8^ Also, LTZ increases the intraovarian androgens, which leads to increased follicular sensitivity to FSH. This leads to expecting a higher ovulation rate with LTZ compared to CC. In the study of Franik et al., ovulation rates were significantly higher with LTZ.^20^ In our study, the ovulation rate in the LTZ group was 76.5% and was significantly higher than the CC group, which was 66.2% (p=0.013). High follicular response positively reflects the clinical success of LTZ.

Legro et al. reported that the higher pregnancy rates with LTZ were related to the elevation of mid-luteal serum progesterone levels.^21^ Progesterone has a crucial role in endometrial decidual alteration and strengthening of the implanted embryo and maintaining pregnancy.^22^ In our study, mid-luteal serum progesterone values were significantly higher in the LTZ group compared to the CC group.

In studies comparing the pregnancy rates and live birth rates of LTZ and CC in the literature, some of the results are similar, and others have superiority to each other. While interpreting these results, it should be kept in mind that each study has strength and limited aspects, and many other variables play a role in pregnancy development. Amer et al. reported that LTZ had higher pregnancy rates (61%) and live birth rates (48.8%) compared to CC (43.0% and 35.4%, respectively). They recommended LTZ as the first line in OI of PCOS patients.^11^ Liu et al. reported that the ovulation rate of LTZ was higher than CC. However, there was no statistically significant difference between pregnancy rates and live birth rates with LTZ and CC.^14^ Therefore, they suggested that CC remains the first-line OI agent in PCOS patients. In this study, the clinical pregnancy rate was 33.1%, and the live birth rate was 29.1% in the LTZ group. In the CC group, the clinical pregnancy rate was 20.9%, and the live birth rate was 17.5%. LTZ was superior to CC in terms of both clinical pregnancy rates and live birth rates.

The abortion rate is higher in PCOS patients than in the normal population^23^ Disorders such as endometrial receptivity, corpus deficiency, impaired quality of ovum, hyperandrogenism, and insulin resistance are among the causes of abortion in patients with PCOS.^14^ Abortion rates were similar in the majority of studies comparing CC and LTZ.^20^ Following the literature, in our study, abortion rates were similar in both groups (p=0.804).

OI with LTZ often results in mono-follicular ovulation. LTZ is thought to have this effect by causing a short FSH window mimicking the menstrual cycle.^11^ This effect leads to a reduction in the risk of multiple pregnancies. In a recent study, mono-follicular development rates and singleton pregnancy rates were higher in PCOS patients with LTZ than CC.^24^ However, in an extensive series of meta-analysis, there was no significant difference between the multiple pregnancy rates of LTZ and CC.^17^ In this study, the mean number of follicles ≥14 mm was similar, and there were no multiple gestations in both groups. However, we think that the lack of multiple pregnancies, especially in the CC group, is due to the low sample size.

A study conducted in 2005 showed high rates of locomotor malformation and cardiac malformation in infants born due to the OI with LTZ caused concern and setback in LTZ use.^17^,^25^ However, many studies conducted after this study showed that the risk of fetal/neonatal anomalies did not increase in LTZ pregnancies when compared with CC.^21^ In our study, there was no statistically significant difference in terms of fetal anomaly rates in both groups. Also, the total fetal anomaly rate was 2.2% in this study. We think that this rate is lower than expected due to both the low sample size and the fact that we do not know if there is any fetal anomaly in the pregnancies that result in abortion.

### Strength and limitaitons of the study

The strengths of this study were that each patient was diagnosed with the same criteria, the demographic characteristics and laboratory values of both groups before treatment were similar, and each patient was treated with the same treatment protocol specific to the group of patients. The limitations of this study were that the retrospective nature of the study, the low number of sample size, and the duration from the beginning of treatment to pregnancy occurrence were not evaluated.

## CONCLUSION

Our study results suggest that both endometrial thickness and ovulation rates were higher in the LTZ group than the CC group. As a result, LTZ is superior to CC in terms of pregnancy rates and live birth rates in PCOS patients. Therefore, we recommend that LTZ should be the first-line treatment agent in patients with PCOS.

### Author’s Contribution:

**MNS:** Conceived and designed the study and is responsible and accountable for the accuracy and integrity of the work.

**MNS & SCO:** Did data collection, statistical analysis, manuscript writing, review and final approval of manuscript.
